# Precision public-health intervention for care coordination: a real-world study

**DOI:** 10.3399/BJGP.2022.0067

**Published:** 2023-01-10

**Authors:** Andre Q Andrade, Jean-Pierre Calabretto, Nicole L Pratt, Lisa M Kalisch-Ellett, Vanessa T Le Blanc, Elizabeth E Roughead

**Affiliations:** Quality Use of Medicines and Pharmacy Research Centre, Clinical and Health Sciences, University of South Australia, Adelaide.; Quality Use of Medicines and Pharmacy Research Centre, Clinical and Health Sciences, University of South Australia, Adelaide.; Quality Use of Medicines and Pharmacy Research Centre, Clinical and Health Sciences, University of South Australia, Adelaide.; Quality Use of Medicines and Pharmacy Research Centre, Clinical and Health Sciences, University of South Australia, Adelaide.; Quality Use of Medicines and Pharmacy Research Centre, Clinical and Health Sciences, University of South Australia, Adelaide.; Quality Use of Medicines and Pharmacy Research Centre, Clinical and Health Sciences, University of South Australia, Adelaide.

**Keywords:** audit and feedback, care coordination, digital health, general practice, intervention study, precision public health, veterans

## Abstract

**Background:**

Health emergencies disproportionally affect vulnerable populations. Digital tools can help primary care providers find, and reach, the right patients.

**Aim:**

To evaluate whether digital interventions delivered directly to GPs’ clinical software were more effective at promoting primary care appointments during the COVID-19 pandemic than interventions delivered by post.

**Design and setting:**

Real-world, non-randomised, interventional study involving GP practices in all Australian states.

**Method:**

Intervention material was developed to promote care coordination for vulnerable older veterans during the COVID-19 pandemic, and sent to GPs either digitally to the clinical practice software system or in the post. The intervention material included patient-specific information sent to GPs to support care coordination, and education material sent via post to veterans identified in the administrative claims database. To evaluate the impact of intervention delivery modalities on outcomes, the time to first appointment with the primary GP was measured; a Cox proportional hazards model was used, adjusting for differences and accounting for pre-intervention appointment numbers.

**Results:**

The intervention took place in April 2020, during the first weeks of COVID-19 social distancing restrictions in Australia. GPs received digital messaging for 51 052 veterans and postal messaging for 26 859 veterans. The digital group was associated with earlier appointments (adjusted hazard ratio 1.38 [1.34 to 1.41]).

**Conclusion:**

Data-driven digital solutions can promote care coordination at scale during national emergencies, opening up new perspectives for precision public-health initiatives.

## INTRODUCTION

There is significant evidence that national emergencies, such as natural disasters or pandemics, result in long-lasting health consequences, including increased mortality months after the initial event.^1^ The consequences disproportionally affect vulnerable populations, such as older people, those who are poor, and people with mental illness.^2^ The COVID- 19 pandemic is an example of a harmful national emergency. Worldwide healthcare utilisation decreased by a third during the pandemic,^3^ because of either direct effect (increased demand) or access restrictions (lockdown measures). It is estimated that current disruptions in health care due to COVID-19 will cause post-pandemic increases in child mortality of up to 44% in low- and middle-income countries.^4^

The COVID-19 pandemic affects older individuals with at least one comorbidity in three different ways:

- the infection is more severe in this population leading to more hospitalisations^5^ and a higher fatality rate;^6^
- the changes induced by the pandemic and its prevention may increase the prevalence of comorbidities, such as mental health conditions;^7^ and
- the impact of national emergencies is the interruption in service provision,^8^ with the potential for a reduced number of healthcare attendances, hospital visits, and laboratory tests.

As a response to the COVID-19 pandemic, the Australian government implemented a series of policies to reduce the risk of widespread infection. Starting on 2 March 2020, the policies included stay-at-home recommendations, where possible, for persons at high risk of poor outcomes if they were to contract COVID-19. To prevent disruptions, the Australian government also implemented a national health plan to maintain access to health services during the pandemic, including options for many medical attendances to be provided by video or telehealth, where appropriate. Video and telehealth Medicare items were available for persons who were at risk of healthcare harms as a result of COVID-19 and in quarantine from 13 March 2020.^9^ Telehealth services were extended to enable vulnerable medical practitioners and health practitioners to provide telehealth for all their patients from 23 March 2020, and further expanded to all practitioners and all patients from 29 March 2020.^10^ Major social distancing restrictions came into effect, including working from home where possible, from the week of 23 March 2020 onwards. Despite the implementation of video and telehealth options, there were concerns that many people may have delayed or avoided their healthcare appointments because of having concerns about catching the virus or not wanting to overload busy doctors. Nationally, a 10% reduction was observed in Medicare services in April 2020 compared with April 2019.^11^

**Table:** How this fits in

Digital technologies hold promise to improve public health, but most initiatives are still limited to electronic health records. A digital, data-driven intervention was developed to promote care coordination for patients vulnerable to poor outcomes of COVID-19. The intervention was delivered across Australia and led to earlier GP appointments when compared with a paper-based version. Similar solutions can be incorporated into emergency preparedness plans to ensure care is coordinated for vulnerable populations.

Given the importance of continuous care provision for patients with chronic diseases, it is the responsibility of public-health professionals to plan and promote strategies to ensure that the needs of patients and caregivers are addressed.^12^ One of the key aspects of care coordination is to identify vulnerable individuals and activate them and their care providers, triggering appropriate action. The use of technology for epidemiological surveillance and intervention development is expected to improve access and equity.^13^ Digital technologies have a growing role in public and preventive health, contributing to the comprehensiveness of care provision;^14^ however, most initiatives are still limited to electronic health records (EHRs) and the use of alerts to drive action.^14^ Effective measures include population-centred interventions using surveillance data,^15^ use of telehealth to improve referral and attendance to mental health clinics,^16^ and decision support to promote preventive actions.^17^

In this article, a real-world, large-scale intervention implemented as a rapid response to the COVID-19 pandemic is described; in addition, the efficacy of delivering the intervention directly to health-provider software digitally, compared with the standard postal delivery, in order to promote visits to the primary care provider, is evaluated.

## METHOD

### Context and setting

In Australia, the GP is the cornerstone of primary care coordination. Approximately 84% of Australians see a GP every year, and 77% of patients have a preferred GP.^18^ The goal of the proposed intervention was to help GPs identify their patients who were vulnerable and to promote follow-up appointments during the period of restrictions. The intervention material was delivered digitally or via the postal service. Some particularities of the Australian health system determined the technology choice, including:

- geographical location — veterans are distributed across Australian states and territories. Although there are a few GPs specialised in veteran care, most GPs have fewer than four veterans under their care. Patients are free to choose their GP, irrespective of geographical location, which may increase patient satisfaction and access, but the lack of patient registration makes it harder for practices to define their population, which has the potential to reduce continuity of care;^19^
- technological readiness — Australian GPs have had near-universal use of EHRs for more than 10 years,^20^ and there is a large penetration of secure-messaging infrastructure for receiving laboratory test results; and
- public-funded, privately operated model of care — primary care in Australia is provided by trained GPs and a universal healthcare scheme, called Medicare, provides basic cover. The Department of Veterans’ Affairs (DVA) provides additional cover for eligible veterans. GPs and primary health clinics are independent providers, so any intervention focused on GPs must be highly collaborative and involve practitioners from the start. There is a high degree of agency during GP appointments, and payers (that is, those funding the care, rather than patients) have limited influence on practice.

### The Veterans’ MATES programme

The initiative reported here was developed as part of the Veterans’ Medicines Advice and Therapeutics Education Services (Veterans’ MATES) programme. Veterans’ MATES is funded by the DVA and aims to improve medicine and health services use and health outcomes for all persons in the veteran community across Australia. The programme drives professional behaviour change via a multifaceted intervention, composed of an educational component and an audit and feedback component delivered to GPs; these are supported by educational components delivered to pharmacists, other relevant health professionals, and veterans. Veterans’ MATES is informed by social cognitive theory,^21^ the transtheoretical model,^22^ and the health-promotion PRECEDE-PROCEED model.^23^ As such, it has a strong focus on education, increasing veterans’ participation in their therapeutic choice, providing repeated interventions over time, and reinforcements and tailored decision support based on available data.

Interventions are created in three sequential steps:

- Step one — an epidemiological inquiry is conducted to identify trends and potential issues in healthcare access and use. Examples include the long-term prescription of medicines recommended for acute issues, doses above guideline recommendations, and lack of screening tests for an eligible population. The programme has access to the DVA’s health claims database, which is updated monthly and includes all dispensed medicines requiring prescription, along with claimed healthcare services and laboratory services, home care, and aged care;
- Step two — educational material, along with audit and feedback documents, are developed. This is a collaborative process with heavy stakeholder involvement, including multiple health professionals and behaviour-change specialists;
- Step three — the identification and delivery of the intervention to veterans and their main healthcare provider. This step requires the use of patient-level information contained in the database to print personalised audit and feedback documents at scale, reaching tens of thousands of veterans and GPs per intervention.

The programme has been extensively described elsewhere,^24^ and has focused on increasing the use of underused medicines, reducing adverse medicine events, reducing use of unnecessary medicines, and improving the utilisation of health services. It has been shown to be effective for changing professional behaviour in different domains,^24^ including promoting medicine review^25^ and osteoporosis screening,^26^ improving uptake of health services,^27^ reducing inappropriate proton pump inhibitor use,^26^ and reducing hypnotics use for insomnia.^28^

Veterans’ MATES ran the first intervention in 2004 and, since then, has delivered four interventions each year. In 2019, a digital precision public-health initiative was implemented, which used digital technology infrastructure available at healthcare provider practices. The goal of this initiative was to use the large longitudinal claims database to create risk profiles, and use data to tailor interventions for GPs. Each digital document sent to GPs is dynamic, and may contain different elements (prompts, goal setting, charts^29^) and recommendations based on identified patient risk. For the intervention to improve care coordination during the COVID- 19 pandemic, the aim was to improve intervention efficacy by increasing GP engagement and reducing the lag between the detection of an issue and the GP being notified about it.

The digital solution developed takes advantage of Australia’s secure-messaging infrastructure, which is commonly used to communicate electronically laboratory results and referral letters from the source directly to the GP’s desktop computer. Electronic documents are visualised in the clinical software, and a GP can request actions from the clinic nurse or reception on the same screen. The paper-based intervention documents were developed as HTML pages, converted to portable document format (PDF), encrypted, and embedded in a Health Level 7 (version 2) file using internally developed software.

### Study design and sample

A non-randomised experimental study was conducted to evaluate the effectiveness of a digital precision public-health intervention at promoting care coordination during national emergencies compared with usual delivery via the postal system.

Patients were allocated to a postal or digital group in two sequential steps. First, eligible patients were identified based on information contained in the administrative health claims database. Patients were eligible if they were identified as being at highest risk of poor outcomes from COVID-19 — that is, aged >70 years with a comorbidity of hypertension,^30^^–^^37^ chronic heart disease,^30^^–^^37^ diabetes,^30^^–^^38^ chronic airways disease,^31^^–^^37^ cerebrovascular disease,^30^^,^^31^^,^^34^^,^^39^ chronic liver disease,^36^ chronic renal failure,^31^^,^^33^^,^^35^^–^^37^ malignancy,^30^^,^^31^^,^^34^^,^^35^^,^^37^^,^^40^ or were immunocompromised.^36^ Identification algorithms were composed of clinical rules with varying levels of complexity, looking for past diagnostic codes (International Classification of Diseases, 10^th^ edition) during hospitalisations, use of medicines indicating treatment for one of the target comorbidities (for example, carvedilol, a medicine that can only be newly prescribed for patients with moderate or severe heart failure in Australia), and combinations of services and medicines used.

After patient eligibility was checked, the primary GP was identified using a proprietary algorithm based on the frequency and recency of appointments. GPs with at least one eligible patient were eligible for inclusion in the intervention. The digital group comprised all GPs with the capability to receive the digital intervention (access to EHR and secure-message delivery); the postal group comprised the remaining GPs.

### Intervention development

The main goal of this intervention was to promote care coordination during lockdown and while social restrictions were in place. It was conducted using a collaborative, pragmatic approach, influenced by Greenhalgh *et al*’s diffusion of innovations model,^41^ in order to develop a solution that could be implemented at national scale. The model summarises a collection of theoretical and empirical findings, and highlights the interplay between an innovation, the adopter, the context in which the innovation takes place, and the implementation and the diffusion processes. The model suggests innovation developers consider nine dimensions during intervention creation:

- innovation;
- adopter;
- assimilation;
- communication and influence;
- system antecedents for innovation;
- system readiness for innovation;
- outer context;
- implementation process; and
- linkage.

**Figure 1.**
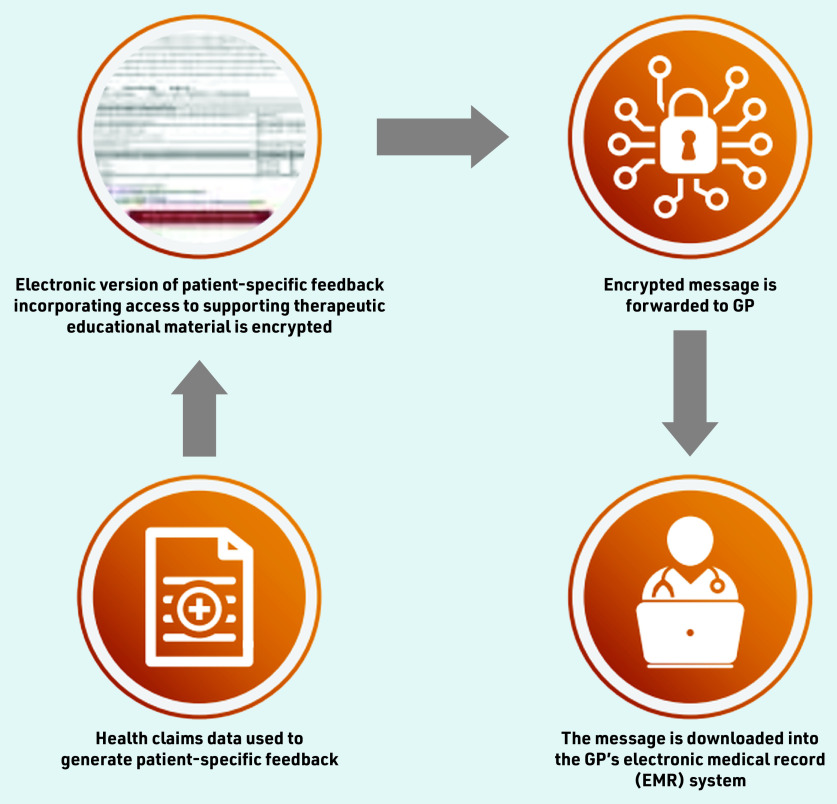
*Schematic representation of the digital intervention delivery.*

The processes involved in intervention development complemented the three steps used by Veterans’ MATES interventions, suited for rapid care coordination (Figure 1). The development of all content and interventions was based on the best evidence available at the time and supported by repeated reviews by panels of health professionals.

**Figure 2.**
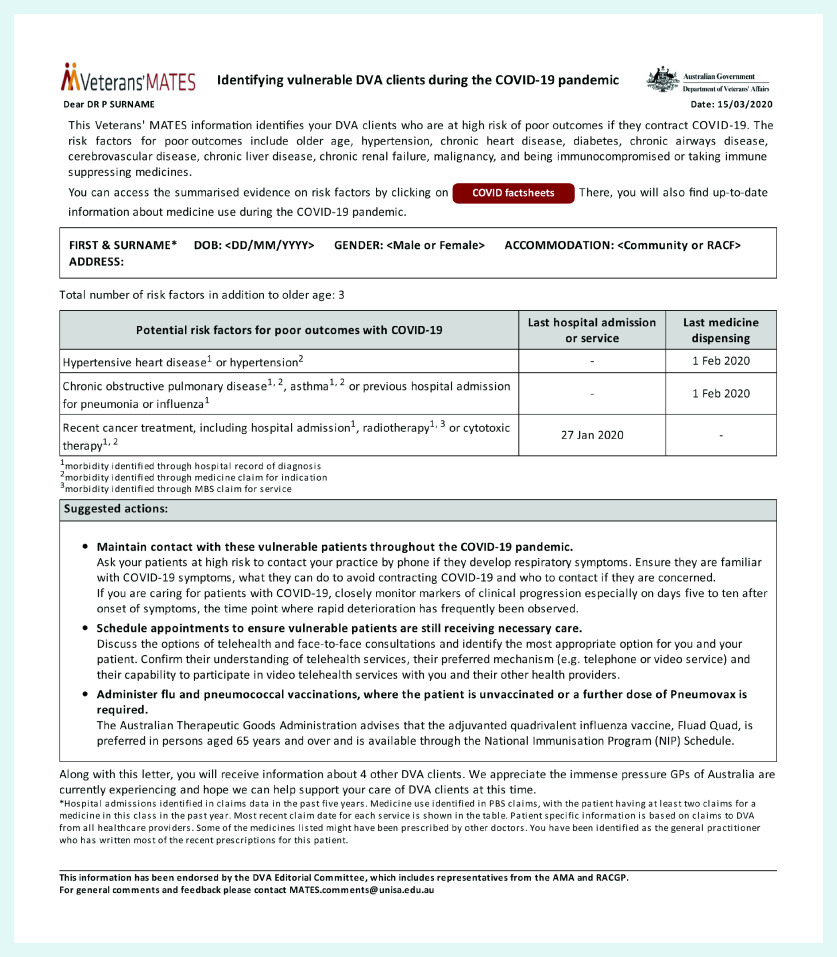
*Sample of the electronic document sent to GPs as part of the intervention.*

The patient-specific feedback document (Figure 2) was developed and submitted to a stakeholder review group, including health professionals (pharmacists and GPs, among others). The behaviour-change techniques (BCTs) used included, as described using the BCT taxonomy:^42^

- goal setting — for example, schedule appointments to ensure vulnerable patients are still receiving necessary care;
- prompts — for example, demonstrate why patient is vulnerable, such as medicine dispensing suggesting respiratory disease; and
- information about health consequences — that is, rationale for suggested actions.

The underlying theory was that the provision of personalised recommendations and BCTs in the form of a document delivered directly to a GP’s clinical software would change that GP’s behaviour, influence patient recall, and trigger an early appointment. The BCTs aim to increase motivation and trigger active patient search by the GP.

In addition to the intervention material delivered to the healthcare practitioner, all veterans living in the community setting (68 872 individuals) received educational material via the postal service. The material included information about COVID-19 infection prevention, prompting them to maintain regular contact with care providers and to continue to adhere to health plans, as well as how to access care during the pandemic given the expansion of video and telehealth appointments, along with free medicine delivery services for eligible persons. The intervention aimed to promote patient activation and the veteran requesting a GP appointment. The online version of the printed materials is available at the Veterans’ MATES website (www.veteransmates.net.au).

### Outcomes and statistical analysis

To evaluate the intervention, the total number of visits post-intervention and the time to first appointment with the primary GP were compared between the digitally delivered and postal delivery groups. It was hypothesised that the time to first appointment would be shorter in the electronically delivered material given improved workflow fit (document is downloaded directly to the clinical software, and appears next to other clinical documents) and the ease of requesting actions in the software. The time to first appointment post-intervention was measured using a time-to-event analysis, in which the index date was the intervention delivery date and the event was first visit with the primary GP. Events were censored at 3 months from the index date.

At the time of the intervention, Australia had in place restrictions on visitors to aged care; as such, veterans living in aged care were excluded from the time-to-event analysis. Cox regression was used to determine whether the time to first GP appointment differed between the intervention delivery modalities after adjusting for gender and patient age at the time of intervention. To account for the difference in GP attendances between the two groups prior to the intervention being delivered, adjustment was made for the number of visits in the previous year. This was done to account for the possibility that those veterans attending clinic with a GP whose practice had electronic clinical management systems had more regular visits to their GP.

Given the large sample and the purposeful sampling, a 99% confidence interval (CI) (*P*<0.01) for all hypothesis tests was considered. All analyses were performed in Python (version 3.7). The main statistical library used for time-to-event was lifelines (version 0.25).

## RESULTS

A total of 77 911 veterans were targeted for the intervention, and 18 577 GPs were identified as their main care provider (mean number of veterans per GP: 4.2 [standard deviation 4.4]). Among those GPs, 61.2% (11 375) were eligible to receive secure-message documents. The 51 052 veterans who had these GPs as their main care providers were included in the digital group. The remaining 7202 GPs were not eligible for digital delivery, and the 26 859 veterans under their care were included in the postal group. Veterans in both groups were similar in age, gender, and geographical distribution (Table 1). However, patients assigned to the digital group had a slightly higher number of usual visits with the primary GP (based on 2019 data, prior to COVID-19 pandemic), despite a similar profile in number of comorbidities (Table 1).

**Table 1.** Baseline comparison between digital and postal groups

	**Digital**	**Postal**
**Veteran participants, *n***	51 052	26 859

**Female (%)**	24 536 (48.1)	12 167 (45.3)

**Average age, years (SD)**	83.96 (9.24)	83.52 (9.34)

**Living in community setting, *n* (%)**	45 759 (89.6)	23 113 (86.1)

**State, *n* (%)**		
**New South Wales**	17 758 (34.8)	9626 (35.8)
**Queensland**	10 723 (21.0)	6626 (24.7)
**Victoria**	10 647 (20.9)	4905 (18.3)
**Western Australia**	5293 (10.4)	2004 (7.5)
**South Australia**	4260 (8.3)	1731 (6.4)
**Australian Capital Territory**	1289 (2.5)	1348 (5.0)
**Tasmania**	980 (1.9)	468 (1.7)
**Northern Territory**	102 (0.2)	151 (0.6)

**Number of comorbidities, *n* (%)**		
**1**	18 243 (35.7)	9772 (36.4)
**2**	16 820 (32.9)	8980 (33.4)
**3**	10 139 (19.9)	5062 (18.8)
**4**	4166 (8.2)	2202 (8.2)
**5**	1359 (2.7)	682 (2.5)
**6**	288 (0.6)	139 (0.5)
**≥7**	37 (0.1)	22 (0.1)

**Average usual number of appointments with primary GP in 2019, *n* (SD)**	10.24 (8.03)	8.13 (8.64)^a^

a P*<.01. SD = standard deviation.*

A total of 51 052 individualised messages were sent, via secure message, to GPs in four sequential batches, starting on 29 April 2020. The remaining 26 859 messages were sent via the postal service to those GPs who were ineligible for secure-message delivery. From the cohort of 51 052 veterans and their GPs targeted for secure message, only four (<0.01%) messages were received from users who advised that they were not the primary care provider of the targeted patient; this suggested that the algorithm for identifying the primary GP was highly accurate.

**Figure 3.**
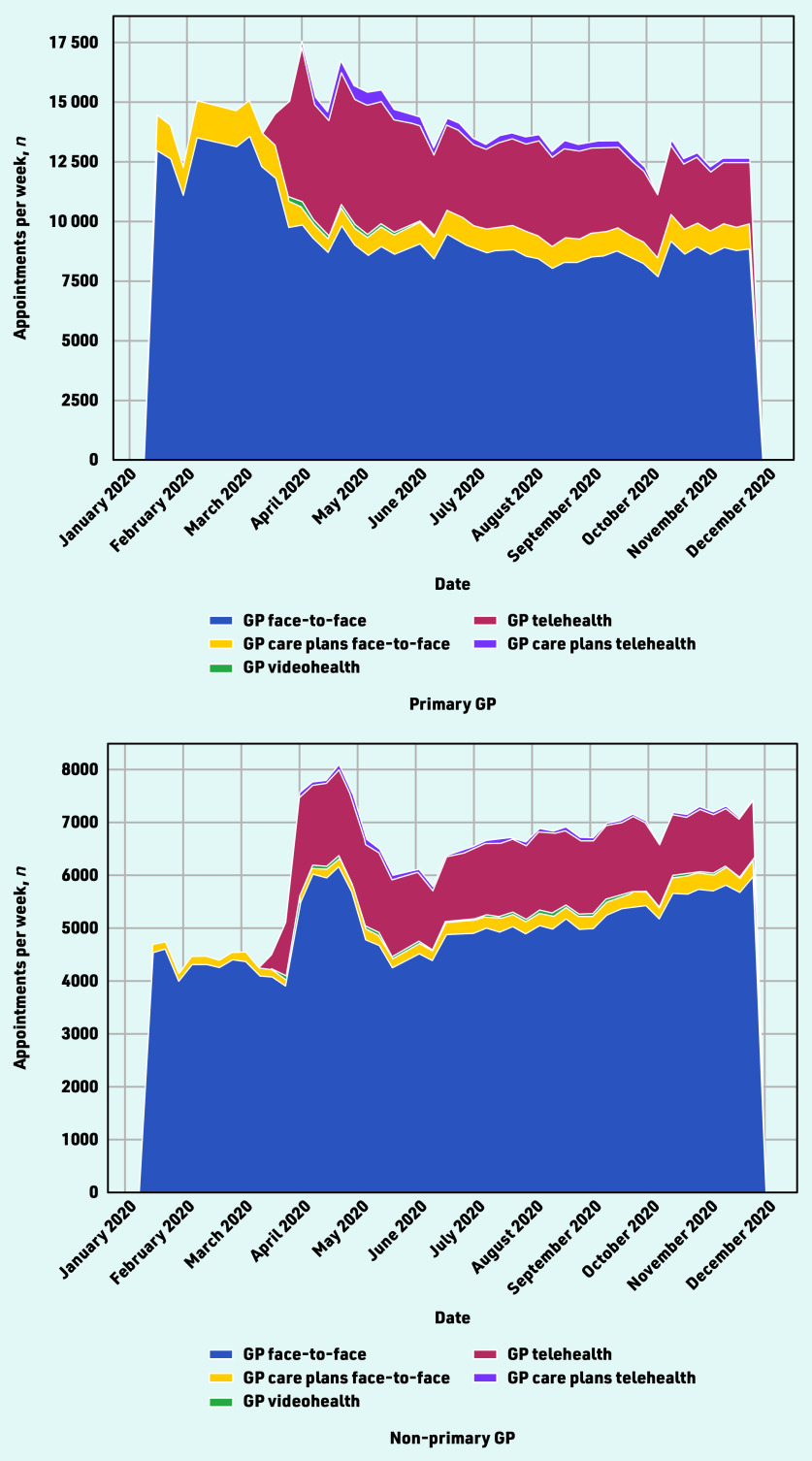
*GP appointments during the first months of the COVID-19 pandemic, by appointment type.*

The total number of appointments with GPs increased substantially over the course of April 2020, from 20 425 visits in the last week of March (25–31 March) to 25 214 in the last week of April (22–28 April). The appointment numbers increased with both the primary GP or other GPs, and was largely driven by services provided by telehealth (Figure 3).

### Time-to-event analysis

**Figure 4.**
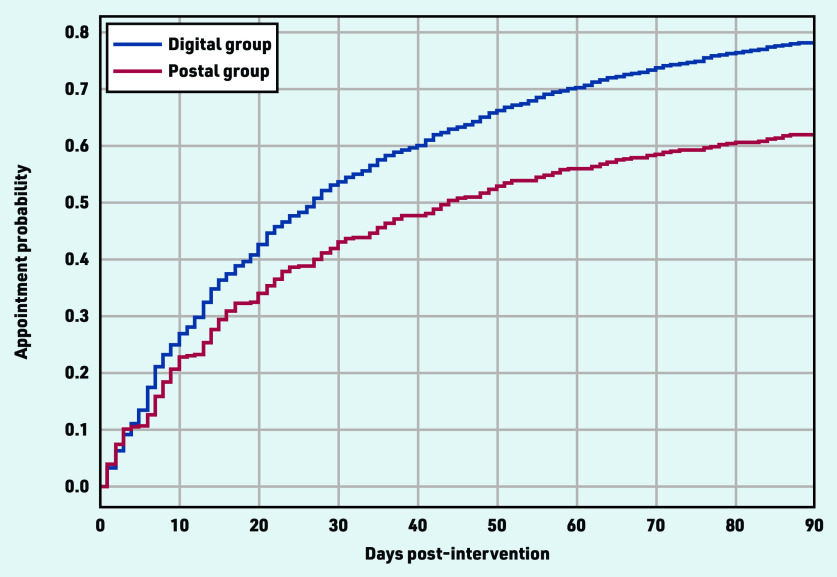
*Kaplan–Meier curve depicting the chance of GP appointment in the first 3 months post-intervention, by group.*

It was found that most targeted veterans living at home (68 872) had at least one appointment with their primary GP (49 833; 72.3%) in the 3 months post-intervention. The chance of patients seeing their primary GP was higher in the digital group (35 607/45 759, 77.8%) than the postal group (14 226 / 23 113, 61.5%), which is reflected in the Kaplan–Meier curve (Figure 4).

At baseline, the digital group had a higher average mean number of visits in the previous year (an indication of the usual frequency) than the postal group (data not shown). To account for the possible influence of the usual number of visits as a determinant of earlier appointment after intervention, the number of appointments in 2019 was included as a confounder; after adjustment, there was a statistically significant difference between the digital and postal delivery groups on time to first primary GP visit (Table 2). Considering individuals with a similar number of visits in the previous year, being in the digital delivery group increased the chance of visiting the primary provider in the 3 months following the intervention when compared with postal group.

**Table 2.** Coefficients of the Cox model

**Covariate**	**Unadjusted hazard ratio (99% CI)**	**Adjusted hazard ratio (99% CI)^a^**
**Postal versus digital group (digital = 1)**	1.50 (1.46 to 1.53)	1.38 (1.34 to 1.41)
**Regular appointments based on the preceding year, *n***		1.04 (1.04 to 1.04)
**Number of comorbidities, *n***		1.09 (1.08 to 1.10)
**Gender (female = 1)**		1.05 (1.03 to 1.08)

a P*<0.001. CI = confidence interval.*

## DISCUSSION

### Summary

This study demonstrated the capacity of a digital intervention to identify vulnerable individuals, reach and engage their main care providers, and measure outcomes. In addition, it was found that the digitally delivered intervention was more effective than that delivered by post at promoting early primary provider visits during the initial months of the COVID-19 pandemic.

### Strengths and limitations

This study benefited from a well-established programme, describing the results of a large-scale intervention at national level. The large sample size increases the confidence in the results and allowed for the stratification of distinct covariate effects in promoting early GP visits.

The main limitation was the lack of a randomised controlled environment to isolate the effects of the intervention alone in promoting GP visits for individuals classified as vulnerable. However, the study results are aligned with a previous randomised controlled trial conducted by the authors, which demonstrated the increased efficacy of a digital intervention in promoting health service utilisation.^43^

The urgency of the situation and the existing system capacity were the paramount issues driving the study design. The comparison with the postal group may have been biased by the purposeful sampling, despite attempts to adjust for the number of usual GP visits in a year. It is possible that the variables affecting access to digital delivery (for example, size of the health provider centre) affected the time to appointment. Several policies were enacted at different times of the COVID-19 pandemic, which may have influenced the results. These include policy changes made by the Australian Department of Health (now the Department of Health and Aged Care), which promoted the use of telehealth by all Australians (including veterans). Telehealth ensured continued access to providers, despite social restrictions, and was sustained throughout the study period. It is possible, however, that the digital group was better set up for telehealth provision.

The COVID-19 pandemic may also have changed patient predisposition to attend their GP appointment, or access telehealth appointments. It is also possible that COVID- 19 changes affected the digital behaviour of physicians, enhancing the effect of the digitally delivered intervention. Furthermore, this intervention took place in the Australian health context, which may have influenced early adoption of digital solutions. Different health systems will need to adapt to local technologies and capabilities.

### Comparison with existing literature

The COVID-19 pandemic promoted a surge in digital health applications for population-level public-health responses: a recent review identified 247 distinct initiatives, ranging from artificial intelligence and big data to diagnose and screen for COVID- 19 infection, to the provision of telehealth for healthcare access.^44^ A common issue with data-driven solutions is scalability and integration into healthcare systems.^45^ In this study, this issue was solved by the clever use of administrative claims database and use of GP secure-messaging infrastructure in large scale.

The study presented here shows that innovative methods of data analysis can be used to extract signals from administrative claims databases, in particular those containing therapeutic information (medicines and services). Access to detailed claims data was key to make possible the identification of high-risk patients and their primary care providers. Claims data have also been successfully used to detect high-risk groups during the COVID-19 pandemic in South Korea.^46^

Further, the study presented here demonstrated that secure-messaging infrastructure can be used to quickly reach primary providers, and digital interventions influenced by behavioural theory are effective in promoting care by the primary provider. Such systems could be used to: guarantee the supply of medicines use for chronic diseases; promote support for patients with mental health conditions; maintain care for persons with time-critical illnesses, such as persons in active bouts of chemotherapy and those undergoing dialysis or requiring home-delivered oxygen; and maintain care of older patients.

An interesting and unexpected finding was the difference in the number of appointments in the year prior to the COVID-19 pandemic between the digital and postal groups. The only criteria separating both groups was access to secure-message delivery. As no other differences between groups were identified, it is possible that the access to technology itself is promoting care provision; however, as shown by the Cox regression and the partial-effect plots, the digital intervention retained its effect even after adjusting for the number of appointments in the previous year. Both findings suggest that access to technology and secure-message delivery should be promoted to clinicians as ways to promote care coordination.

### Implications for practice

Digital infrastructure, coupled with innovative solutions, enables the promotion of care coordination at scale, opening new perspectives for precision public-health initiatives. As well as their importance in usual public-health contexts, the results suggest that solutions using existing digital infrastructures can be useful in emergency-preparedness systems, adding to the list described in Whitelaw *et al*.^47^ Experiences after national emergencies and disasters recognise their impact on patients with chronic diseases and the importance of quickly reacting to healthcare needs of these patients when designing plans.^48^^,^^49^ Given that responses can vary according to the emergency and conditions, the capacity to identify patients with particular comorbidities may prevent negative consequences affecting patients who are vulnerable, such as those with cancer^50^ or severe kidney disease.^51^

This study was one of the first nationwide programmes to use centralised health information to support GPs in caring for patients who were vulnerable during the COVID-19 pandemic. In Australia, involving GPs is key in maintaining adequate care. Given their distributed geographical location and the multitude of clinical software in use, adequate targeting was only possible because of the existence of mature claims databases, which are routinely updated and contain useful clinical information. The results suggest a wider collaboration between public-health officials and GPs could improve efficiency and direct the use of health resources towards patients in need.

This study reinforces the value of developing solutions fit for context using iterative and participatory processes. Finding the right level of complexity of any digital health intervention is particularly susceptible to context change.^52^ Health systems based on consumer-focused health care will benefit from solutions focused on patient activation, such as self-screening tools.^53^ The authors of the study presented here took advantage of secure-messaging infrastructure, which was already incorporated into the clinician workflow. Additionally, the existence of standards (Health Level 7 and PDF) and the availability of structured computer coding libraries provided the required flexibility, speed, and power to develop the intervention. The authors also profited from having strong stakeholder relationships and stakeholder reference groups that had continuously met since the inception of the Veterans’ MATES programme in 2004. The time from conception to full implementation was less than 4 weeks, which included obtaining the required approvals and having clinical and stakeholder review prior to implementation. The stakeholder and clinical goodwill that had been achieved because of engagement with Veterans’ MATES over time, meant that a team of people was available to review materials at short notice, including on-the-ground practitioners who felt they were being bombarded with information in the early days of the pandemic. This same structure was maintained after this study, which allowed the digital intervention to be continuously incorporated into the Veterans’ MATES programme. All eligible GPs currently receive the intervention digitally directly to their clinic software.

Future research will investigate how the intervention can be further personalised, profiting from the capacity to create dynamic documents and use the internet for additional education and enhanced practice auditing. It will also evaluate intervention effectiveness on clinical outcome measures, drawing on long-term longitudinal data.

This study demonstrated that digital delivery (via a secure-messaging infrastructure) promoted visits to the primary GP, and is one of the first to show the feasibility and increased efficacy of a digital intervention to coordinate care at national level during emergencies. Similar solutions can be adapted as a response to emergency events to ensure the care continuation of populations who are vulnerable.

## Data

The data that support the findings of this study are available from the Australian Government’s Department of Veterans’ Affairs; however, restrictions apply to the availability of these data, which were used under licence for the current study, and so they are not publicly available. Access to data should be requested to the data custodian.
